# Living healthier for longer: Comparative effects of three heart-healthy behaviors on life expectancy with and without cardiovascular disease

**DOI:** 10.1186/1471-2458-9-487

**Published:** 2009-12-24

**Authors:** Wilma J Nusselder, Oscar H Franco, Anna Peeters, Johan P Mackenbach

**Affiliations:** 1 Department of Public Health, Erasmus MC, University Medical Center Rotterdam, PO Box 2040, 3000 CA Rotterdam, Rotterdam, The Netherlands; 2 University of Warwick, Warwick Medical School, Health Sciences Research Institute, Coventry, CV4 7AL, UK; 3 Department of Epidemiology and Preventive Medicine, Monash University Central and Eastern Clinical School, Melbourne, Australia

## Abstract

**Background:**

Non-smoking, having a normal weight and increased levels of physical activity are perhaps the three key factors for preventing cardiovascular disease (CVD). However, the relative effects of these factors on healthy longevity have not been well described. We aimed to calculate and compare the effects of non-smoking, normal weight and physical activity in middle-aged populations on life expectancy with and without cardiovascular disease.

**Methods:**

Using multi-state life tables and data from the Framingham Heart Study (n = 4634) we calculated the effects of three heart healthy behaviours among populations aged 50 years and over on life expectancy with and without cardiovascular disease. For the life table calculations, we used hazard ratios for 3 transitions (No CVD to CVD, no CVD to death, and CVD to death) by health behaviour category, and adjusted for age, sex, and potential confounders.

**Results:**

High levels of physical activity, never smoking (men), and normal weight were each associated with 20-40% lower risks of developing CVD as compared to low physical activity, current smoking and obesity, respectively. Never smoking and high levels of physical activity reduced the risks of dying in those with and without a history of CVD, but normal weight did not. Never-smoking was associated with the largest gains in total life expectancy (4.3 years, men, 4.1 years, women) and CVD-free life expectancy (3.8 and 3.4 years, respectively). High levels of physical activity and normal weight were associated with lesser gains in total life expectancy (3.5 years, men and 3.4 years, women, and 1.3 years, men and 1.0 year women, respectively), and slightly lesser gains in CVD-free life expectancy (3.0 years, men and 3.1 years, women, and 3.1 years men and 2.9 years women, respectively). Normal weight was the only behaviour associated with a reduction in the number of years lived with CVD (1.8 years, men and 1.9 years, women).

**Conclusions:**

Achieving high levels of physical activity, normal weight, and never smoking, are effective ways to prevent cardiovascular disease and to extend total life expectancy and the number of years lived free of CVD. Increasing the prevalence of normal weight could further reduce the time spent with CVD in the population.

## Background

Cardiovascular disease (CVD) is the number one cause of mortality and morbidity in the world, contributing largely to health care expenditures, and lost productivity due to disability and death [1]. The effects of modifiable risk factors, such as smoking, physical inactivity and overweight on CVD onset and death are well-established [2-10]. Promoting heart-healthy behaviors is a major strategy to reduce the development of CVD [11]. It may also reduce the number of years lived with cardiovascular disease in the population, but this is not necessarily true. Heart-healthy behaviors, such as non-smoking and physical activity reduce mortality not only from CVD but also from other causes [2,4], and if the same intervention not only delays cardiovascular disease but also death, the time spent with cardiovascular disease may decrease or increase [12]. To prioritise between preventive interventions that promote heart healthy behaviours, comparable information on the number of years without and with CVD is needed.

Previous studies suggest that non-smoking [13], normal weight [14] and high/moderate levels of physical activity [15] each are associated with a longer CVD-free and total life expectancy, but to a different extent. The effects on the number of years lived with cardiovascular disease also appeared to vary between these behaviours. Normal weight reduced [14] and never smoking [13] increased the time spent with cardiovascular disease, whilst physical activity [15] did not suggest a clear association. However, there were a number of methodological differences between the studies so it is unclear to what extent these results reflect real differences in the potential effects.

We aimed to calculate in a comparable way the effects of never smoking, normal weight and physical activity in populations aged 50+ on transitions from no CVD to cardiovascular disease, no CVD to death, and cardiovascular disease to death and on their life expectancy and the number of years lived with and without cardiovascular disease.

## Methods

### Data Sources

The Framingham Heart Study consisted of a cohort of 5209 respondents residing in Framingham, Massachusetts, between 1948 and 1951. The study included both men (n = 2336) and women (n = 2873) aged 28 to 62 years. The cohort has been examined biannually for over 50 years. Further description can be found in [16] and on the FHS website http://www.framinghamheartstudy.org/. Ethical approval was not required as this study was based on secondary data analysis.

### Study Sample

We split the FHS dataset into three, non-overlapping follow-up intervals of 12 years. We chose 12 years of follow-up to maximize power and minimize the risks for selection and reverse causation, which means that lower physical activity levels, BMI levels, and quitting smoking are caused by ill health and not the other way around [15,17]. Moreover, information on physical activity was available for a limited number of exams. The follow-up intervals started therefore at the exams 4 (1956-1958), 11/12 (1969-1973) and 19/20 (1985-1989). Each interval started with a measurement of the health behaviors and confounders (education was only measured once). Using the pooling of repeated observations method [18], the three intervals were pooled, yielding a total of 9773 observation-intervals. The same participant may thus be followed during three periods until the event (CVD or death) occurs or the subject is censored because of competing events or lost to follow-up (n = 64). Splitting and pooling of non-overlapping intervals does not affect the number of events and person-years, but allows updating of health behavior status and confounders in each interval and coverage of the whole age range of age 50 and over. Persons below age 50 at follow up (n = 208) were excluded, yielding a total of 9565 observation intervals. Information on physical activity was missing for 592 observation-intervals. These were not excluded, but grouped into a separate category as missing physical activity. Information on one or more other health behaviors was missing for 81 observation-intervals, on the presence of baseline diseases for 179 observation-intervals and on blood pressure for 1 observation-interval. After excluding those with missing data (in total 261 observation intervals) we included 4209 participants starting at exam four, 3446 at exam 11/12 and 1649 at exam 19/20 yielding a total of 9304 observation-intervals from 4634 subjects.

### Assessment of physical activity, smoking, and BMI

For physical activity, participants were asked to estimate how long they spent in a typical day at various levels of activity: sleeping, resting, or engaged in light, moderate and heavy physical activity. The reported levels of activity were weighted based on the estimated oxygen consumption for each activity to reflect metabolic expenditure correspondent to metabolic equivalents (METs). Weights used were: for sleeping 1, for being sedentary 1.1, for light activity 1.5, for moderate activity 2.4 and for heavy activity 5. Finally, a daily physical activity score was calculated by adding the sum of the weighted hours for each level of activity. The minimum possible score is 24 for a participant sleeping 24 hours/day. Further detail on the assessment of physical activity and calculation of the daily physical activity score can be found elsewhere [15,19]. Based on tertiles of the physical activity score, we grouped the participants into three levels: low (<30), moderate (30-33) and high (>33) physical activity level[15,17]

For smoking, subjects were classified as never, ever and current smoker at the start of each of the three follow-up periods [13].

Four categories of body mass index (BMI) were defined: <18.5 kg/m^2 ^for underweight, 18.5 ≤ BMI<25 kg/m^2 ^for normal weight, 25 ≤ BMI<30 kg/m^2 ^for overweight, and ≥ 30 kg/m^2 ^for obesity [20]. We separated those with underweight from the normal weight group to avoid reverse causation, but will not present results for this small group.

### Assessment of Cardiovascular Disease

The primary outcome of our study is first incident or fatal cardiovascular disease and death. Cardiovascular disease included coronary heart disease (angina, coronary insufficiency, myocardial infarction and sudden or not sudden death as consequence of coronary disease), congestive heart failure, stroke, transient ischemic attack and intermittent claudication. A panel of three physicians evaluated all events; agreement of all three was required. For all events (incidence of CVD and death) exact dates were recorded. More detail on the evaluation of outcomes in the Framingham Heart Study is available elsewhere [21]

### Measurement of potential confounders and intermediates

Potential confounders considered were: age, sex, education (eight grade or less/higher than eighth grade), marital status (single/married/widowed/separated or divorced), co-morbidity at each baseline (having at least one of the following diseases: cancer, diabetes, left ventricular hypertrophy, arthritis, ankle edema or any pulmonary disease) and the exam at the start of follow-up (exam 4, 11/12 or 19/20). The latter was included to correct for potential cohort and period effects, since the participants could belong to three different periods of follow-up and different birth cohorts. Depending on the health behaviour of interest, smoking, physical activity, BMI and blood pressure (hypotension, normotension, pre-hypertension, and hypertension) were considered as confounders. Normotension was defined as systolic blood pressure (SBP) between 90 and 120 mmHg and diastolic blood pressure (DBP) between 50 and 80 mmHg, pre-hypertension as SBP between 120 and 139 mmHg and DBP between 80 and 89 mmHg and hypertension as SBP above 140 or DBP greater or equal than 90 mmHg and hypotension as SBP below 90 and DBP below 50 [22]. Data on cholesterol levels were not available for more than 40% of the participants and was therefore not included.

### Data Analysis

We built population-based multi-radix multi state life tables [23] with three states (free of CVD, history of CVD and death) to assess associations between each of the three heart health behaviors (smoking, physical activity, and BMI), and life expectancy with and without CVD at age 50. We first derived overall transition rates by single year of age and sex, irrespective of risk factor status, for three transitions ('no history of CVD' to 'history of CVD', or to 'death' and from 'history of CVD' to 'death') which together determine life expectancy with(out) CVD. Next we calculated hazard ratios (HRs) to assess the relation between the risk factor status and the three transitions. Finally these HRs, in combination with the overall transition rates and risk-factor prevalence were used in a multi-state life table to calculate life expectancy with and without CVD at age 50 by risk factor level.

The overall transition rates were specific for age and sex. Prevalence of health behavior level was assessed by sex, 10-year age groups, and separately for subjects with and without cardiovascular disease.

Overall age-specific transition rates were calculated with Poisson regression using the Gompertz distribution to obtain smoothed age-specific rates. HRs adjusted for sex, age, and potential confounders were also based on this Poisson regression model. We assumed proportional hazards, unless the log-likelihood ratio test showed a significant improvement of the fit of the model (significance level 5%) when interactions with age (50 to 70 years vs. older than 70), or sex were included. This was only true for the sex-smoking interaction for the transition from non-CVD to CVD. Three final models were selected. One model adjusted for only age and sex, the second model adjusted additionally for confounders, and the third model also for intermediates. Co-morbidity and exam at the start of follow up were included as confounders. Education and marital status were not included, as these factors did not substantially alter the HRs of the health behaviours. Additionally, physical activity, BMI and blood pressure were included as confounders for smoking, and smoking as confounder for physical activity and BMI. We did not include BMI as confounder for physical activity and vice versa, as these factors can act as both confounders and/or intermediate factors in the causal pathway and correcting would then imply ignoring part of their causal effect. Nonetheless, correcting for BMI in assessing the effects of physical activity, or vise versa, hardly changed the HRs of BMI and physical activity, respectively. The same was true for correcting for blood pressure in assessing the effects of non-smoking.

Blood pressure was considered as an intermediate factor for both BMI and physical activity as blood pressure is in the causal chain between the BMI/physical activity and cardiovascular disease and death [14].

We re-ran all analyses excluding the first two years of follow-up to avoid reverse causation, but as this hardly affected the HRs they were not excluded in the main analyses.

We then built multi-state life tables based on the HRs, overall transition rates and prevalence of the specific health behavior. Two alive states were included: no history of CVD and history of CVD. We used HRs adjusted for age, sex and potential confounders. The life tables started at age 50 and closed at age 100. No back flows were allowed and only the first entry into a state was considered. Similar calculations have been described previously [15,17]. All statistical analyses were done using STATA version 8.2 for Windows (Stata Corporation, college station TX, USA, 2003). Life tables were calculated in Excel, worksheets are available upon request. We calculated confidence intervals for all life expectancies and their differences using Monte Carlo simulation (parametric bootstrapping) [24] with @RISK (Anonymous 2000; MathSoft Inc 1999, http://www.palisade.com/RISK/), 10000 runs.

## Results

### Baseline characteristics

The mean age of the pooled study population was 62 years, 57% were women, 76% were married and 74% had an educational level higher than 8th grade (Table 1). This pooled study population is the aggregate of observations in three intervals, which together capture all ages in the population aged 50 and over. The second and third period included increasingly older ages and more recent years, and thus included a higher percentage of women, unmarried (widowed) persons, persons with co-morbidity and lower percentage of smokers as compared to the first one.

**Table 1:** Baseline characteristics of the Framingham Heart Study population in exam 4, exam 11/12, exam 19/20 and in the pooled dataset

	Exam 4	Exam 11/12	Exam 19/20	Pooled
**Characteristics**	**N = 4209**	**N = 3446**	**N = 1946**	N = 9304*

Age, mean (SD), y	50.5(8.2)	63.7 (7.9)	74.9 (6.3)	59.6(12.1)
Women, No. (%)	2346 (56)	1990 (58)	1004 (61)	5340 (57)
Marital status, No. (%)^a^				
Single	366 (9)	244 (7)	121 (7)	731 (8)
Married	3589 (85)	2449 (73)	945 (57)	6983 (76)
Widowed	167 (4)	564 (17)	534 (32)	1265 (14)
Divorced/Separated	87 (2)	93 (3)	49 (3)	229 (2)
Education, No. (%)^b^				
8^th ^grade or less	1178 (29)	847 (25)	339 (21)	2364 (26)
Higher than 8^th ^grade	2942 (71)	2500 (75)	1264 (79)	6706 (74)
Pa level, No. (%)				
Low	1294 (31)	979 (28)	446 (27)	2719 (29)
Moderate	1611 (38)	1155 (34)	426 (26)	3192 (34)
High	1017 (24)	1121 (33)	734 (45)	2872 (31)
Missing	287 (7)	191 (6)	43 (3)	521 (6)
Smoking Status, No. (%)				
Never smoker	1521 (36)	1176 (34)	604 (37)	3301 (35)
Former smoker	466 (11)	1055 (31)	807 (49)	2328 (25)
Current smoker	2222 (53)	1215 (35)	238 (14)	3675 (39)
Any co-morbidity†, No. (%)	1351 (32)	1471 (43)	1190 (72)	4012 (43)
BMI^‡^, mean (SD), kg/m^2^	25.9 (4.1)	26.1 (4.2)	26.4 (4.5)	26.1 (4.2)
SBP, mean (SD), mmHg	133.9 (22.9)	141.3 (22.2)	143.7 (22.1)	138.3 (22.7)
DBP, mean (SD), mmHg	83.7 (12.2)	97.2 (29.8)	75.9 (10.9)	87.3 (22.0)

Abbreviations: Pa, Physical activity; BMI, Body Mass Index; SBP, Systolic Blood Pressure; DBP, Diastolic Blood Pressure.

*Pooled observation intervals of 4634 subjects alive and with no missing data on baseline chronic diseases and on smoking, hypertension and BMI.

† Any co-morbidity: cancer (n = 447 in pooled population, diabetes mellitus (n = 536), left ventricular hypertrophy (n=536), arthritis (n=2088), ankle edema (n=1168) or pulmonary disease (n = 755)

‡ Body Mass Index, calculated as BMI = weight in kg/height^2 ^in m^2^

^a^Based on: 4109 (exam 4), 3350 (exam 11/12), 1649 (exam 19/200) and 9208 subjects (pooled dataset).

^b^Based on: 4120 (exam 4), 3347 (exam 11/12), 1600 (exam 19/200) and 9070 subjects (pooled dataset).

### Risk of Cardiovascular Disease and Death

#### Onset of cardiovascular disease

High physical activity, never smoking (only men), and normal weight were each associated with 20-40% lower risks of developing CVD (Table 2) as compared to low physical activity, current smoking and obesity. Overweight and former smoking were also associated with lower risks of CVD compared to obesity and current smoking. The effects of smoking differed significantly by sex, for women the effects were of a smaller magnitude and non-significant. Adjustment for confounders and intermediates generally slightly attenuated the effects. Only in women adjustment slightly increased the effects of smoking (Table 2).

**Table 2:** Hazard ratios for the different transitions for men and women, based on the Framingham Heart Study based on the pooled dataset

	No CVD to CVD			No CVD to death			CVD to death		
Number of events	1698			739			1465		
Person years	65685.47			65685.47			18139.31		

	**Model 1** **HR (95% CI)** **Adj. for age+sex**	**Model 2** **HR (95% CI)** + confounders*	**Model 3** **HR (95% CI)** **+ intermediates†**	**Model 1** **HR (95% CI)** **Adj. for age+sex**	**Model 2** **HR (95% CI)** + confounders*	**Model 3** **HR (95% CI)** **+ intermediates†**	**Model 1** **HR (95% CI)** **Adj. for age+sex**	**Model 2** **HR (95% CI)** + confounders*	**Model 3** **HR (95% CI)** **+ intermediates†**

Physical activity									
Low	1		1	1	1	1	1	1	1
Mod	0.93 (0.82;1.05)	0.95 (0.84;1.07)	0.98 (0.87;1.10)	0.75 (0.63;0.91)	0.77 (0.64;0.92)	0.78 (0.65;0.93)	0.93 (0.82;1.05)	0.96 (0.85;1.09)	0.98 (0.86;1.11)
High	0.75 (0.66;0.85)	0.78 (069;0.89)	0.81 (0.71;0.92)	0.68 (0.56;0.82)	0.69 (0.57;0.85)	0.71 (0.58;0.86)	0.67 (0.58;0.77)	0.74 (0.64;0.85)	0.76 (0.66;0.87)
Smoking Men									
Current	1	1	1	1	1	1	1	1	1
Former	0.74 (0.60;0.91)	0.73 (0.59;0.90)	0.73 (0.59;0.90)	0.54 (0.44;0.66)	0.56 (0.46;0.68)	0.56 (0.46;0.68)	0.76 (0.66;0.88)	0.77 (0.67:0.90)	0.77 (0.67:0.90)
Never	0.66 (0.56;0.77)	0.63 (0.54;0.74)	0.63 (0.54;0.74)	0.67 (0.56;0.81)	0.69 (0.57;0.83)	0.69 (0.57;0.83)	0.73 (0.64;0.83)	0.82 (0.71;0.94)	0.82 (0.71;0.94)
Smoking Women									
Current	1	1	1	1	1	1	1	1	1
Former	0.92 (0.78;1.09)	0.83 (0.70;0.99)	0.83 (0.70;0.99)	0.54 (0.44;0.66)	0.56 (0.46;0.68)	0.56 (0.46;0.68)	0.76 (0.66;0.88)	0.77 (0.67:0.90)	0.77 (0.67:0.90)
Never	0.93 (0.76;1.14)	0.89 (0.72;1.08)	0.89 (0.72;1.08)	0.67 (0.56;0.81)	0.69 (0.57;0.83)	0.69 (0.57;0.83)	0.73 (0.64;0.83)	0.82 (0.71;0.94)	0.82 (0.71;0.94)
BMI									
Obese	1	1	1	1	1	1	1	1	
Overweight	0.86 (0.75;0.98)	0.86 (0.75;0.98)	0.92 (0.81;1.05)	0.81 (0.65;1.01)	0.80 (0.64;1.00)	0.83 (0.66;1.04))	0.93 (0.80;1.08)	0.93 (0.80;1.08)	0.96 (0.83;1.11)
Normal weight	0.69 (0.60;0.79)	0.69 (0.59;0.79)	0.79 (0.69;0.92)	0.99 (0.79;1.22)	0.95 (0.76;1.18)	0.99 (0.79:1.23)	1.10 (0.95;1.29)	1.10 (0.94;1.27)	1.13 (0.97;1.31)

*Corrected for (co) morbidity (any of the following diseases: cancer, diabetes mellitus, left ventricular hypertrophy, arthritis, ankle edema or any pulmonary disease), and the start of follow-up (exam 4, 11/12 or 19/20). Physical activity is additionally corrected for smoking. Smoking is additionally corrected for physical activity, BMI and hypertension, and BMI is additionally corrected for smoking.

† Corrected for (co) morbidity (any of the following diseases: cancer, diabetes mellitus, left ventricular hypertrophy, arthritis, ankle edema or any pulmonary disease), and the start of follow-up exam 4, 11/12 or 19/20), and all other health behaviors and blood pressure. For smoking this is the same model as model 2.

#### Death in persons without CVD

The risk of dying in those without a history of CVD was 30% lower among persons with high physical activity levels and never smokers, as compared to persons with low levels of physical activity and current smokers (Table 2). Moderate physical activity and former smoking were also associated with reductions in the risk of mortality. Adjustment for confounders and intermediates slightly attenuated the effects (Table 2). Normal weight and overweight were not associated with lower mortality risks as compared to obesity.

#### Death in persons with CVD

The risk of dying once cardiovascular disease is present was lower among never smokers (men) and those with high levels of physical activity (Table 2). Adjustment for confounders and other health behaviours slightly attenuated the effects. Having normal weight or overweight as compared to being obese, and moderate physical activity as compared to low, did not significantly alter the mortality risks once CVD is present.

### Life Expectancy with and without cardiovascular disease

Never smokers, persons with high physical activity, and persons with normal weight had higher total and CVD-free life expectancy than current smokers, persons with low physical activity and obese persons, respectively, but the magnitude of the effects differed (Table 3). Never smoking was associated with the largest gains in total life expectancy (4.3 years in men and 4.1 years in women), followed by high physical activity (3.5 and 3.4 years, respectively), and finally by normal weight (1.3 and 1.0 years respectively). The largest gains in CVD-free life expectancy were also found for never smoking (3.8 and 3.4 years, respectively) although differences were only slightly larger than for high physical activity and normal weight. Gains in CVD-free life expectancy were similar for high physical activity (3.0 years, 3.1 years, respectively) and normal weight (3.1 and 2.9 years, respectively). Former smokers, persons with moderate physical activity and persons with overweight also had higher total and CVD-free life expectancy than current smokers, persons with low physical activity and obese persons, but generally the differences were smaller than in the groups with the healthiest behaviours (Table 3).

**Table 3:** Total life expectancy (total LE), CVD-free life expectancy (LE free of CVD) and life expectancy with CVD (LE with CVD), and difference, in Years at Age 50, men and women

	Total LE (yrs)	Dif Total LE (yrs)	LE free of CVD (yrs)	Dif LE free of CVD (yrs)	LE with CVD (yrs)	Dif LE with CVD (yrs)
Men						
Physical activity†						
Low	26.4 (25.7;27.3)	Ref	19.7 (18.9;20.6)	Ref	6.7 (6.2;7.3)	Ref
Moderate	27.7 (26.8;28.8)	1.3 (0.3;3.2)	20.8 (19.7;22.0)	1.1 (0.0;2.2)	6.9 (6.2;7.7)	0.2 (-0.6;0.9)
High	30.0 (29.0;31.0)	3.5 (2.5;4.6)	22.8 (21.7;23.9)	3.0 (1.8;4.3)	7.2 (6.5;8.1)	0.5 (-0.3;1.4)
Smoking*						
Never	29.7 (28.6;30.7)	4.3 (3.0;5.5)	22.6 (21.3;24.0)	3.8(2.1;5.5)	7.0(6.2;7.8)	0.4 (-0.6;1.5)
Former	29.4 (28.2;30.5)	4.0 (2.1;5.6)	23.3 (21.7;25.0)	4.5(2.0;6.8)	6.0 (5.2;6.9)	-0.6 (-2.0;0.9)
Current	25.4 (24.4;26.4)	Ref	18.8(17.8;20.0)	Ref	6.6 (5.8;7.4)	Ref
BMI†						
Normal	28.1 (27.4;28.9)	1.3 (0.2;2.5)	22.1 (17.7;20.4)	3.1(1.9;4.4)	6.0(5.5;6.5)	-1.8 (-2.8;-0.9)
Overweight	28.5 (27.7;29.3)	1.6 (0.3;3.1)	20.8 (19.9;21.6)	1.7 (0.2;3.3)	7.7(7.1;8.4)	-0.1 (-1.3;1.1)
Obesity	26.8 (25.6;28.1)	Ref	19.0 (17.7;20.4)	Ref	7.8(6.9;8.8)	Ref
Women						
Physical activity†						
Low	32.7 (31.9;33.5)	Ref	26.3 (25.5;27.1)	Ref	6.4 (5.9;7.0)	Ref
Moderate	34.1 (33.2;35.1)	1.5 (0.5;2.5)	27.6 (26.6;28.7)	1.3 (0.2;2.4)	6.6 (5.9;7.3)	0.2(-0.6;0.9)
High	36.1 (35.0;37.2)	3.4 (2.3;4.5)	29.4 (28.2;30.6)	3.1 (1.9;4.3)	6.7 (5.9;7.5)	0.3 (-0.5;1.1)
Smoking*						
Never	34.5 (33.9;35.2)	4.1(3.1;5.2)	28.1 (27.5;28.8)	3.4(2.7;4.1)	6.3 (5.9;6.8)	0.7 (0.2;1.3)
Former	33.2 (32.2;34.2)	2.8 (1.3;4.3)	27.0 (26.1;27.8)	2.2 (1.2;3.3)	6.2 (5.7;6.8)	0.6 (-0.2;1.4)
Current	30.4 (29.3;31.5)	Ref	24.7 (23.9;25.7)	Ref	5.6 (5.0;6.3)	Ref
BMI†						
Normal	33.8 (33.1;34.6)	1.0 (-0.2;2.2)	28.2 (27.4;29.0)	2.9(1.6;4.2)	5.6 (5.1;6.1)	-1.9 (-2.9;-1.0)
Overweight	34.5 (33.7;35.4)	1.7(0.2;2.3)	27.1 (26.22;28.1)	1.9 (0.3;3.5)	7.4 (6.7;8.1)	-0.2 (-1.5;1.0)
Obesity	32.8 (31.6;34.1)	Ref	25.2 (24.0;26.5)	Ref	7.6 (6.7;8.6)	Ref

† Corrected for (co) morbidity (any of the following diseases: cancer, diabetes mellitus, left ventricular hypertrophy, arthritis, ankle edema or any pulmonary disease), and the start of follow-up (exam 4, 11/12 or 19/20), and smoking

*Corrected for (co) morbidity (any of the following diseases: cancer, diabetes mellitus, left ventricular hypertrophy, arthritis, ankle edema or any pulmonary disease), and the start of follow-up (exam 4, 11/12 or 19/20), physical activity, BMI and blood pressure.

Normal weight was the only heart-healthy behavior associated with a decrease in the number of years with CVD (1.8 years (men) and 1.9 years (women)) (Table 3). In contrast, women who never smoked spent 0.7 more years with CVD than current smokers.

## Discussion

Never smoking, high levels of physical activity, and normal weight at age 50 and over appears to be not only associated with increases in total life expectancy, but also with a greater number of years lived free of CVD. The effects on total life expectancy (both sexes) and years lived free of CVD (men) appeared greatest for never smoking; although for women the increase in years free of CVD differed less between the three health hearty behaviors. Normal weight was the only behavioral factor in our study found to also reduce the time lived with CVD.

Never smoking (men), high levels of physical activity, and normal weight were each associated with lower risks of developing CVD as compared to low physical activity, current smoking and obesity, respectively. Never smoking and high levels of physical activity also reduced the risks of dying in those with and without a history of CVD, but normal weight did not. The greater number of years lived free of CVD among those with high levels of physical activity or never smoking is due to their lower incidence of CVD, in combination with lower mortality without a history of CVD. The greater number of years free of CVD and reduction in time lived with CVD among those with a normal weight is due to lower incidence of CVD, not being accompanied with a reduction in mortality.

The HRs we found support existing evidence that non-smoking, physical activity and normal weights reduces incidence of CVD [2,4,8]. The reductions in mortality among persons without cardiovascular disease associated with never smoking and physical activity, are also in line with documented effects for a large set of fatal diseases, including respiratory diseases and several cancers [2,4]. Our results also confirm that non-smoking and physical activity reduces mortality in persons with a history of CVD [25,26]. The effects of BMI on mortality are more disputed, and appear to depend on the age considered [27]. The lack of a harmful effect of overweight and obesity on mortality in elderly persons with CVD, is in agreement with the study of Kalanar-Zadey[28]. It is noteworthy that our findings on mortality in persons with and without CVD are not in contrast with existing evidence that obesity increases overall mortality. In fact, our findings suggests that higher total mortality among obese persons is mainly due to higher rates of onset of CVD, which, in combination with higher mortality risks among persons with CVD, results in higher total mortality, and not due to worse survival.

Our results show smaller differences in total life expectancy at age 50 associated with BMI compared to prior analyses also based on the Framingham Heart Study [14,29] Additional analyses comparing the studies in more detail (data available on request) suggested that the majority of the difference was explained by the fact that whilst the current analysis assessed the effect of BMI in a population age 50 and over and updated BMI information every 12 years, the prior analyses assessed BMI only at age 40-50, and followed this cohort without updating this information to more recent years and ages. The effects of BMI on mortality have been shown to be smaller in elderly populations [27]. We found also smaller differences associated with smoking as compared to prior work based on the Framingham Heart Study [13], which compared never and always smokers, instead of never, current and former smokers in the present study.

Some limitations of our study must be considered. This is a life table analysis based on a prospective observational study where no intervention was performed. Therefore it has the inherent weaknesses of all cohort studies and lacks the strength of causality that randomized trials could offer. Reverse causation, which means that lower physical activity levels, BMI levels, and quitting smoking are caused by ill health and not the other way around, is an important issue to consider since it could introduce bias in the evaluation of the effect of these health behaviors. While owing to the longitudinal design we fulfill the temporality criteria of Bradford and Hill [30] that the effect (CVD onset or death) has to occur after the cause (unhealthy behavior), we can not rule out that the health behavior is affected by the health of the individual. Different approaches exist to reduce the effect of reverse causation but there is no method to eliminate it completely. To correct for reverse causation we adjusted our analyses for co-morbidities at baseline instead of excluding the subjects with disease at the start of follow-up, since we are interested in the effect of health behaviors in the general elderly population and not in selected healthy populations. Finally we examined whether excluding the first two years of follow up did reduce the HRs, but found minimal effect (data not shown).

Another limitation is that during the Framingham Heart Study, physical activity and smoking were evaluated by self-report, which might be affected by recall bias. In contrast height and weight were measured in the study. Differences in measurement of these behaviors would hamper our comparison if the resulting misclassification affects the transitions differently (e.g. affect transition from cardiovascular disease to death, but not from non-CVD to cardiovascular disease, or death). However, misclassification, if present, is likely to be non-differential and to affect each transition similarly.

Participants included in the FHS were mainly white; therefore, our results may not apply to other ethnic groups. Furthermore, the historical character of the FHS, limits the extrapolation of findings to today's populations. Nonetheless by focusing on those over age 50, and updating BMI, smoking and physical activity information every 12 years, we have automatically put more weight on contemporary data (as older age in the cohort means more recent years). Life expectancy at age 50 in our study is very similar to the life expectancy at age 50 in the US in 2000-2004 (life expectancy at age 50: men: FHS: 27.3, US 28.4; women: 33.7 and 32.2 years respectively)[31]. Also, our study focuses on comparisons between different risk factor categories (e.g. normal weight vs. obesity).

The key strengths of our study include using a well-organized historic cohort that has been followed biannually for decades (we used 36 years), and provides upgraded information on health behaviours, covariates and outcomes. Further, this is the first comparative life table analysis of the effects of the main heart-healthy behaviours on life expectancy and life expectancy with and without CVD.

## Conclusion

Our study suggests that interventions increasing population levels of physical activity, non-smoking, and normal weight are an effective way to reduce the incidence of cardiovascular disease, postpone mortality and to extend both total life expectancy and the duration of life lived free of CVD. Whilst trends in non-smoking are moving in the right direction, the associated benefits in cardiovascular disease prevention run the risk of being obscured by reverse trends in the prevalence of obesity and physical inactivity [32]. If obesity levels continue to rise we would expect the years lived with cardiovascular disease in the population to increase, regardless of any advances in non-smoking and physical activity prevalence.

## Competing interests

The authors declare that they have no competing interests.

## Authors' contributions

WN as guarantor of this article accepts full responsibility for the integrity of the data and the accuracy of the data analysis, had full access to all the data in the study, and controlled the decision to publish. *Study concept and design*: WN, OF, AP, and JM. *Acquisition of data*: WN, OF and AP. *Analysis and interpretation of data*: WN, OF and AP. *Drafting of the manuscript*: WN. *Critical revision of the manuscript for important intellectual content*: OF, AP, and JM. *Statistical analysis*: WN. *Obtained funding*: JM and WN. *Study supervision*: JM. All authors read and approved the manuscript.

## Pre-publication history

The pre-publication history for this paper can be accessed here:

http://www.biomedcentral.com/1471-2458/9/487/prepub
